# Distributed Optimal Random Access Scheme for Energy Harvesting Devices in Satellite Communication Networks

**DOI:** 10.3390/s19010099

**Published:** 2018-12-28

**Authors:** Pengxu Li, Gaofeng Cui, Weidong Wang

**Affiliations:** 1 School of Electronic Engineering, Beijing University of Posts and Telecommunications, Beijing 100876, China; cuigaofeng@bupt.edu.cn (G.C.); wangweidong@bupt.edu.cn (W.W.); 2 Information and Electronics Technology Lab, Beijing University of Posts and Telecommunications, Beijing 100876, China

**Keywords:** satellite communication networks, random access, energy harvesting, distributed optimal policy

## Abstract

This paper considers satellite communication networks where each satellite terminal is equipped with energy harvesting (EH) devices to supply energy continuously, and randomly transmits bursty packets to a geostationary satellite over a shared wireless channel. Packet replicas combined with a successive iteration cancellation scheme can reduce the negative impact of packet collisions but consume more energy. Hence, appropriate energy management policies are required to mitigate the adverse effect of energy outages. Although centralized access schemes can provide better performance on the networks’ throughput, they expend extra signallings to allocate the resources, which leads to non-negligible communication latencies, especially for the satellite communication networks. In order to reduce the communication overhead and delay, a distributed random access (RA) scheme considering the energy constraints is studied. Each EH satellite terminal (EH-ST) decides whether to transmit the packet and how many replicas are transmitted according to its local energy and EH rates to maximize the average long-term network throughput. Owing to the nonconvexity of this problem, we adopted a game theoretic method to approximate the optimal solution. By forcing all the EH-STs to employ the same policy, we characterized and proved the existence and uniqueness of the symmetric Nash equilibrium (NE) of the game. Moreover, an efficient algorithm is proposed to calculate the symmetric NE by combining a policy iteration algorithm and the bisection method. The performance of the proposed RA scheme was investigated via numerous simulations. Simulation results showed that the proposed RA scheme is applicable to the EH devices in the future low-cost interactive satellite communication system.

## 1. Introduction

In recent years, the packet-oriented Internet protocol (IP) traffic and machine-to-machine (M2M) applications supported by satellite communication networks have received increasing attention in the research community, which is significant for complementing the terrestrial infrastructure to provide global seamless coverage [1]. For emerging applications, satellite networks need to efficiently tackle bursty traffic with a low duty cycle generated by a massive number of terminals, with constrain energy and limit computational resource. However, this presents a significant challenge for the multiple access protocols in satellite communication networks. Traditional solutions for resource allocation based on fixed assignment and demand assignment are obviously not applicable to this type of traffic [1]. Random access (RA) protocols have become a candidate multiple access scheme in interactive satellite networks, because they are insensitive to the network size and traffic characteristics, as well as easy to be implemented for satellite terminals. Specifically, RA is a kind of distributed uplink access scheme. Terminals upload their data packets directly via a shared wireless channel, and they do not need to request resources. For bursty traffic, it is inefficient for massive terminals to request resources frequently, especially for the network made up by a geosynchronous orbit (GEO) satellite. Moreover, centralized resource allocation and scheduling for massive terminals are really complicated and impractical, and the extra communication delay brought by resource allocation is intolerant. Thus, RA can be well applicable to the large network size and bursty traffic.

Aloha and slotted Aloha (S-Aloha) are the classical RA protocols, and they are widely applied in satellite communication networks [2]. However, their performance in terms of network throughput is really poor due to packet collisions. Satellite communication networks suffer from large propagation delay, especially for the geostationary satellite. Packet collisions decrease the throughput of the network and further enlarge the access delay caused by packet retransmission, which is intolerant to interactive applications. Therefore, the research for more efficient satellite RA schemes is triggered by this issue. Thanks to the evolution and implementation of a physical layer forward error correction (FEC) coding scheme and iterative signal processing [3] at the gateway station, the performance of random access is improved greatly, which is more practical for interactive satellite networks. Contention resolution diversity slotted Aloha (CRDSA) [4,5] as a candidate RA scheme for the next generation of interactive satellite systems [6] adopted the concept of time diversity and successive interference cancellation (SIC) to enhance the performance of RA, where the maximum throughput could reach up to 0.55 packets/slot. More specifically, a packet is transmitted two or more times within a frame, and each packet contains the location information of the replicas in the packet header, which is used to resolve the collisions. CRDSA promotes the research on Aloha-based RA schemes, especially for the satellite communications scenario. Indicatively, some variants of CRDSA were presented in References [7,8,9,10]. These RA schemes inherited the concept of time diversity by changing the packet repetition rate or combining with suitable coding schemes to further improve the throughput. Moreover, some RA protocols that adopted the train of thought of CRDSA but without time synchronization were researched in References [11,12,13]. The asynchronous mechanism makes the received packets probably collide partially with a specific packet. Consequently, the throughput of the network can be further boosted by adopting powerful FEC coding schemes, which are used to recover the packets with limited interference. These enhanced RA protocols exploited packet replicas to recover the collided packets, but transmitting extra replicas consumed more energy.

Recent advances in energy harvesting (EH) technologies enable the devices supplying sustained energy by collecting energy from the surrounding environment, e.g., solar, wind, heat, etc. [14]. Many systems, such as wireless sensor networks (WSNs) and satellite communication networks, can take advantages of EH technologies [15]. Satellite terminals equipped with EH devices can execute tasks such as data acquisition and data transmission autonomously over a long period of time. Therefore, energy management for the satellite terminals is one of the significant and realistic issues in the RA procedure. Moreover, due to the unpredictable and random energy harvested, the design of appropriate RA schemes considering energy management is essential to minimize the negative impact of energy outage and to optimize the long-term network overall throughput. Unfortunately, the EH process and the problem of energy efficiency of the satellite terminals were not taken into consideration in the aforementioned satellite RA schemes.

Various multiple access schemes with optimal energy management policies in terrestrial networks have been researched in the literature [16,17,18,19]. In Reference [16], authors studied the tradeoff between throughput and the delay of a sensor node with an EH source. The authors of [17] proposed a policy to determine whether the EH device should transmit the data or not, according to its current energy level, so as to maximize the utility of the networks. The authors of [18,19] characterized the stable region in the scenario of two bursty nodes with EH capability randomly accessing a common receiver. These studies are suitable for only one or two terminals, which are not realistic in the satellite networks.

The design of medium access control (MAC) protocols that can support multiple EH sensors was presented in References [20,21,22,23], focusing on time division multiple access (TDMA), framed Aloha, and dynamic framed Aloha. An EH contention tree-based access (EH-CTA) protocol was addressed in Reference [24], which exploited a tree-splitting algorithm to recover collisions and considered the energy availability. The authors of [25] put forward an EH aware reservation dynamic framed slotted Aloha (EH-RDFSA) protocol for the case of wireless M2M networks. The above works [20,21,22,23,24,25] are considered in the terrestrial EH-WSNs scenario, and the access policies are decided by a central controller according to the energy levels of each EH sensor. The centralized schemes are expected to achieve the best performance because packet collisions can be avoided by proper management at the central controller. However, this kind of centralized policies is only appropriate for relatively small scales of networks [26]. For satellite communication networks, the number of EH satellite terminals (EH-STs) is much larger than that of sensor nodes in terrestrial EH-WSNs, and the energy state information uploaded by massive EH-STs to the central controller in every slot will lead to unaffordable communication overhead. Furthermore, the resource allocation procedure will cause additional communication delay, which is unacceptable in satellite communication networks, especially for the networks made up by geosynchronous orbit (GEO) satellites.

Some studies on multiple access schemes with distributed energy management policies were presented in References [27,28,29]. In this respect, a distributed dynamic optimal policy which maximized the sum throughput by adjusting each EH node’s transmission power was introduced in Reference [27]. A decentralized access scheme was designed in Reference [28], where each EH node decided to transmit or discard a packet according to the packet’s utility and the energy level independently by a game theoretic analysis method. A distributed scheduling scheme adopting an iterative technique to find the efficient rate and power scheduling for boosting the average throughput was proposed in Reference [29].

The terrestrial RA schemes with energy management policies mentioned above are based on pure S-Aloha, where no contention resolution mechanisms are applied to improve the performance of the networks. The S-Aloha scheme suffers a high collision probability, especially for the heavy traffic load. Without contention resolution mechanisms, colliding frequently requires a large number of retransmissions, which yields very large latencies in the satellite networks. Therefore, those RA schemes based on pure S-Aloha cannot be applied to the satellite scenario directly. In addition, the population of the satellite terminals in the satellite coverage range is much larger than that in the terrestrial cell, which makes the RA schemes with centralized energy management policies unpractical. Thus, the design of RA schemes with efficient energy management policies for the interactive satellite communication networks is urgent.

Driven by these requirements, this paper introduces a distributed optimal RA scheme based on the CRDSA protocol for EH-STs in satellite communication networks. Unlike some asynchronous and spread spectrum based RA protocols, such as enhanced spread spectrum Aloha (E-SSA) [30] and minimum mean square error enhanced spread spectrum Aloha (ME-SSA) [31], CRDSA is a non-spread spectrum protocol but requires time slot synchronization. The reason for choosing CRDSA as the basic protocol is its robustness and simplicity of implementation compared with spread spectrum systems [12]. Moreover, CRDSA is a candidate RA protocol for the next generation of interactive satellite systems [6], which is interested in practice. The main contributions of this paper can be summarized as:

- Propose a distributed optimal RA scheme in satellite communication networks with the consideration of energy management policies by extension of the CRDSA protocol towards an EH scenario. Each EH-ST determines whether to transmit the packet and how many replicas are sent according to its local energy information so as to maximize the average long-term network throughput;
- develop an analytical model of the average long-term throughput with constraints of packet loss ratio and energy. A game theoretic method is adopted to tackle the nonconvex optimization problem. By employing the same access scheme to all EH-STs, the symmetric Nash equilibrium of this game is characterized, and its existence and uniqueness are proved;
- exploit a policy iteration algorithm combined with a bisection method to approximate the optimal solution of the game. The performance of the proposed RA scheme by both numerical analysis and extensive simulations is investigated under different EH rates for different metrics as throughput, packet loss ratio, and data delivery probability.

The rest of this paper is organized as follows. Section 2 introduces the system model and performance metrics. Problem descriptions and formulation are presented in Section 3. Section 4 analyzes and solves the optimization problem. Simulation results are shown in Section 5. Finally, Section 6 concludes this paper and indicates future work.

*Notations*: Uppercase boldfaces, lowercase boldfaces and normal letters denote the matrices, vectors and scalars respectively, such as X, x and *x*.

## 2. System Model

**Figure 1:**
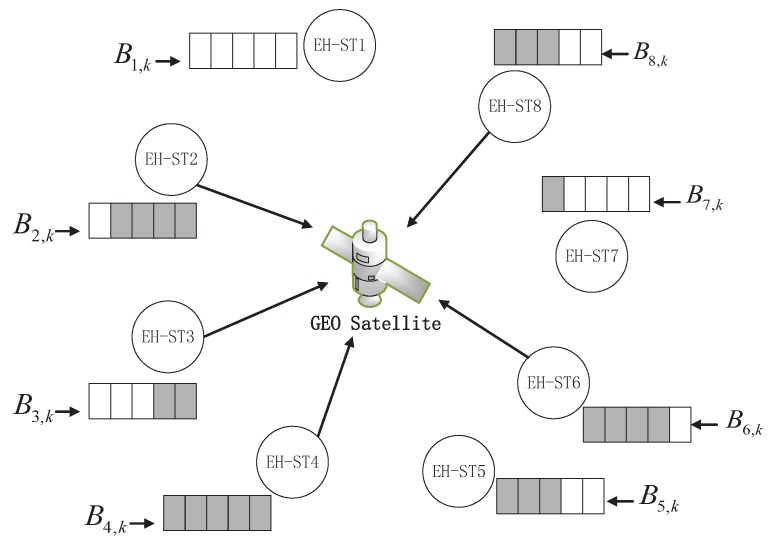
Satellite communication networks with energy harvesting terminals.

The scenario under consideration in this paper consists of *U* wireless EH-STs, where EH-STs send data packets via a shared wireless channel to a GEO satellite, as shown in Figure 1. These EH-STs are placed in the outdoor field to monitor the environment and post the acquired data to the satellite [32]. The data packets received by the satellite are forwarded to the ground gateway station transparently with independent backhaul channels, which are assumed to be error free. The energy harvested by each EH-ST from the environment is stored in a rechargeable battery for the radio frequency unit and sensing apparatus. Each EH-ST is equipped with a processing unit to manage the available energy. The following parts of this section introduce the data model, MAC protocol, energy consumption, energy storage, and energy harvesting models of the system in detail.

### 2.1. MAC Protocol Operation and Performance Metrics

**Figure 2:**
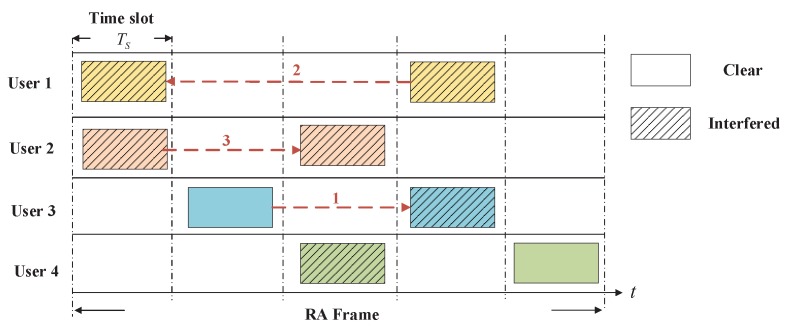
Contention resolution diversity slotted Aloha (CRDSA) frame structure and interference cancellation procedure (Packet replica l=2).

(1) MAC Protocol Operation: The adopted MAC protocol is based on the idea of CRDSA and its frame structure is shown in Figure 2 [4]. Time is slotted, and each RA frame consists of *N* time slots. The duration of each time slot is assumed to be equal to the packet transmission time, which is denoted as $T_{s}$. Once the EH-STs have registered in the network, they will keep time synchronization via the procedures described in Reference [33] or in Reference [34]. An EH-ST can transmit only one MAC packet per RA frame. In fact, the EH-ST will transmit *l* replicas of the same MAC packet physically, where $l\in Z^{+}$ and l≤N. These replicas carry the same preamble and payload information and are randomly put in *l* slots of an RA frame. For a specific replica, the payload should contain the location information of other replicas within the frame. Once a packet is detected successfully at the gateway station (e.g., the first packet of user 3 in Figure 2), it will use the location information to cancel the interference caused by other replicas on other slots (e.g., the second packet of user 3 in Figure 2). Most of the packets that are collided initially can be recovered by iterating the above approach.

For packets decoded at the gateway station, it is assumed that channel information is always available, which can be acquired by the methods mentioned in References [4,10]. For every frame, the EH-STs will always have a packet to send, and all packets are with equal importance. Each EH-ST decides whether to transmit the packet independently following the policy ω, which is only related to its current energy level and EH rate. Specifically, ω indicates two parts. One is the number of packet replicas to send, which is denoted as *l*, and the other one is the probability of transmitting these replicas, which is denoted as η. For simplicity, this paper only considers stationary policies independent of each frame, and $\omega =\left(\omega_{1},\omega_{2},\ldots ,\omega_{U}\right)$ denotes as the joint transmission policy of the network. If the packet is not transmitted at the current frame, or it fails to be transmitted successfully, it will be transmitted in the next frame.

(2) Performance Metrics: The performance of the RA scheme is measured by the packet loss ratio (PLR), the throughput *T* of the system, and data delivery probability $p_{d}$, where PLR and *T* are related to the normalized traffic load *L*, number of packet replicas *l*, and number of time slots in a frame *N* [4]. Similar to the definition of throughput in Reference [10], for given *l* and *N*, which are usually predefined in the network, the relationship between PLR and *T* is defined as:

(1) $$T\left(L,l,N\right)=\left(1-PLR\left(L,l,N\right)\right)L,$$

where L=M/N, and *M* is the number of the co-transmitted EH-STs within the duration of one frame ($M\in Z^{+}$). Moreover, PLR is supposed to be a continuous increasing function with respect to (w.r.t) the traffic load *L* [4], and the traffic load increases with the increase of packet transmission probability.

The data delivery probability is the probability of the data of any EH-ST *u* to be successfully delivered and correctly received by the satellite during the *k*th frame when there are new packets ready to be reported at the start of the frame *k*. This metric, denoted as $p_{d}$, measures the ability of the RA scheme to successfully transmit the packets from EH-STs to the satellite in every frame, without depleting their energy. $p_{d}$ is expressed as:

(2) $$\begin{array}{c} p_{d}\left(k\right)=\mathrm{Pr}\;\left[u\;\mathrm{transmits}\;\mathrm{successfully}\;\mathrm{in}\;\mathrm{frame}\;k|u\;\mathrm{has}\;\mathrm{new}\;\mathrm{data}\;\mathrm{in}\;\mathrm{frame}\;k\right]. \end{array}$$

Note that the successfully transmitted packets by EH-ST *u* in Equation (2) include both the noncollided packets from the beginning and the packets that have been resolved during its frame time. Moreover, the statistical probability (Equation (2)) is made by each EH-ST independently. Since EH technologies can provide EH-STs with potentially perpetual operation, the average long-term data delivery probability can be expressed as $p_{d}^{LT}=\lim_{k\to \infty }p_{d}\left(k\right)$.

Packet delay is another metric to describe the average delay of a packet that is successfully received at the gateway station. Each EH-ST transmits *l* packet replicas with the probability η independently. If there is not enough energy in the battery, it will wait until it has enough energy to transmit the packets. The evolution of the available energy in the battery can be modeled as a Markov chain, which is introduced in detail in Section 3. Moreover, if a packet fails to be received at the gateway station, it will be retransmitted with the same policy ω, according to the residual energy. Therefore, the packet delay $D_{pk}$ is measured as the number of frames that elapse from the start of one frame where the packet was transmitted for the first time, until the end of the frame where the packet was received successfully. According to the description above, the packet delay distribution can be expressed as [35]:

(3) $$\mathrm{Pr}\;\left(D_{pk}=f\right)=\left\{\begin{array}{c} \sum_{e=0}^{e_{max}}\pi_{\eta }\left(e\right)\eta \left(e\right)\left(1-PLR\right),\;\;\;\;\;\;\;\;\;\;\;\;\;\;\;\;\mathrm{for}\;f=1\\ \sum_{e=0}^{e_{max}}\{\left[\pi_{\eta }\left(e\right)\eta \left(e\right)PLR\right]\cdot \left[(\pi_{\eta }\left(e\right)\eta \left(e\right)\;\cdot \;\left(1-PLR\right))\right]\\ \;\;\;\;\times {\left[\left(1\;-\;\pi_{\eta }\left(e\right)\eta \left(e\right)\right)\;+\;PLR\;\cdot \;\pi_{\eta }\left(e\right)\eta \left(e\right)\right]}^{f-2}\},\;\;\;\;\;\mathrm{for}\;f>1 \end{array}\right.,$$

where $\pi_{\eta }\left(e\right)$ is the steady-state probability of the available energy *e*, and the derivation of $\pi_{\eta }\left(e\right)$ is presented in the next section. η(e) is the packet transmission probability and $e_{max}$ is the capacity of the battery. Specifically, the term $\pi_{\eta }\left(e\right)\eta \left(e\right)\left(1-PLR\right)$ represents that the packet is received successfully. For f>1, the term $\pi_{\eta }\left(e\right)\eta \left(e\right)PLR$ means the packet is transmitted in the first frame, but it is not received by the gateway station. Furthermore, the term ${\left[\left(1\;-\;\pi_{\eta }\left(e\right)\eta \left(e\right)\right)\;+\;PLR\;\cdot \;\pi_{\eta }\left(e\right)\eta \left(e\right)\right]}^{f-2}$ can be explained as that the packet is still not received in the next f−2 frame periods due to the energy shortage or packet collisions. Thus, the long-term average packet delay $D_{pk}^{LT}$ is given by:

(4) $$D_{pk}^{LT}=\sum_{f=1}^{\infty }f\cdot \mathrm{Pr}\;\left(D_{pk}=f\right).$$

### 2.2. Energy Consumption and Storage Models

In this paper, the rechargeable battery of each EH-ST is modeled as a buffer [36], and each position in the buffer can hold one energy unit. It is assumed that packets are transmitted with equal power, which will consume one energy unit for transmitting one physical packet in a frame, and the other energy expenditures due to battery leakage or data collecting, etc. are not taken into consideration. The number of energy units that are stored in a battery of an EH-ST is denoted by $E\in \left\{0,1,2,\ldots ,e_{max}\right\}$, where $e_{max}$ represents the capacity of the battery. Denote the amount of energy units of EH-ST *u* at the frame *k* as $E_{u,k}$, where $k\in Z^{+}$, and the evolution of $E_{u,k}$ is formulated as follows:

(5) $$E_{u,k+1}=min\left\{E_{u,k}-l_{u,k}\cdot Q_{u,k}+B_{u,k},e_{max}\right\},$$

where $Q_{u,k}$ is the packet transmission action of EH-ST *u*. $Q_{u,k}=1$ if the EH-ST determines to transmit the current packet with $l_{u,k}$ replicas, which will consume $l_{u,k}$ energy units, otherwise the EH-ST *u* will remain idle for $Q_{u,k}=0$. $B_{u,k}$ represents the amount of energy that the EH-ST *u* harvested within the duration of frame *k*, and the harvested energy within the duration of frame *k* can only be used for the next frame. Thus, if there is no energy left in the battery, then the EH-ST *u* will remain idle ($Q_{u,k}=0$).

### 2.3. Energy Harvesting Model

The communication scenario under consideration in this paper is mainly applied to environmental monitoring, and each EH-ST can harvest different amounts of energy units from the ambient energy sources. For instance, for a solar source, EH-STs in the direct sunlight situation will harvest more energy than those in a cloudy situation. Therefore, we assume the energy harvester of EH-ST *u* acquires $B_{u,k}$ energy units from the environment within the duration of frame *k*. Based on the energy harvesting models mentioned in References [22,24,25], the energy arrival process in this paper is assumed to follow Poisson distribution during one frame transmission time with parameter $b_{k}$, which represents the stochastic and intermittent nature of the ambient energy sources. Thus, the probability of $B_{u,k}$ energy units arrived within the duration of frame *k* is:

(6) $$\beta \left(B_{u,k},b_{k}\right)=\frac{b_{k}^{B_{u,k}}exp\left(-b_{k}\right)}{B_{u,k}!}.$$

The energy arrival process of the components of $B_{k}=\left(B_{1,k},B_{2,k}\ldots ,B_{U,k}\right)$ is considered to be identically and independently distributed (i.i.d) over time and all EH-STs. $\bar{\beta }$ denotes the average energy units harvested within a long period of time, which reflects the average long-term EH rate, and $\bar{\beta }=E\left[B_{u,k}\right]$.

## 3. Problem Descriptions and Optimization

This paper focuses on designing a distributed RA policy for EH-STs in satellite communication networks. Following this way, all the EH-STs in the networks are considered to have their own local information, i.e., the energy level at the current time and the EH rate. Therefore, EH-ST *u* decides whether to transmit the packet in the current frame *k* or keep idle based only on $E_{u,k}$, independent of the energy level of other EH-STs ($E_{i,k},i\neq u$). For the given $E_{u,k}$, the probability of EH-ST *u* transmitting its current packet ($Q_{u,k}=1$) with $l_{u,k}$ replicas is $\eta \left(E_{u,k}\right)$, which is the transmission policy of our design.

Supposing there are *M* EH-STs transmitting packets in the same frame duration, given the initial state of energy levels $E_{0}=e_{0}\in E^{U}$, the policy ω, the number of packet replicas *l*, and the number of time slots in a frame *N*, the average long-term throughput contributed by the specific EH-ST *u* can be denoted as follows:

(7) Tωue0=limK→∞1K∑k=0K−11−PLRM,l,Nηu,k·UM×∏i≠u,jηi,k∏j≠u,i1−ηj,k∣e0,ω,

where *i* indicates the other co-transmitted EH-STs in addition to EH-ST *u*, and *j* represents the rest of the EH-STs which keep idle. The term $1\;-\;PLR\left(M,l,N\right)$ represents the throughput contributed by EH-ST *u*, and the term UM∏i≠u,jηi,k∏j≠u,i1−ηj,k is a binomial operator which stands for the probability of *M* EH-STs transmitting packets. Specifically, the average probability of EH-ST *u* transmitting $l_{u,k}$ replicas in energy level *e* is denoted as:

(8) $$\eta_{u}\left(e\right)=\mathrm{Pr}\left[Q_{u,k}=1|E_{u,k}=e\right].$$

Furthermore, the expected throughput contributed by EH-ST *u* in energy level *e* is denoted as $g\left(\eta_{u}\left(E_{u,k}\right)\right)$, which is given by:

(9) $$g\left(\eta_{u}\left(E_{u,k}\right)\right)=E\left[1-PLR\left(M,l,N\right)|E_{u,k}=e\right].$$

$g\left(\eta_{u}\left(e\right)\right)$ is a concave function w.r.t the traffic load, which is related to the transmission probability $\eta_{u}\left(e\right)$. Therefore, Equation (7) can be restated as:

(10) Tωue0=limK→∞1K∑k=0K−1gηuEu,kηu,k·UM×∏i≠u,jηi,k∏j≠u,i1−ηj,k∣e0,ω.

Moreover, the average long-term throughput of the network is defined as the number of the packets successfully uploaded to the satellite, which can be represented as:

(11) $$T_{\omega }\left(e_{0}\right)=\sum_{u=1}^{M}T_{\omega }^{\left(u\right)}\left(e_{0}\right).$$

This paper aims to design the packets transmission policies ω to maximize the throughput of the network, i.e.,

(12) $$\omega^{*}=\underset{\omega }{arg max}T_{\omega }\left(e_{0}\right).$$

However, the design of transmission policies ω for the EH-STs in the satellite communication networks needs to consider the following trade-offs:

- More packet replica EH-STs transmitting seems to provide a better performance of packet detection [7]; nonetheless, too many replicas will cause channel saturation easily in the heavy traffic load [35] and battery depletion occurs more frequently;
- larger transmission probability makes EH-STs transmit more often, which yields large access latencies due to packet collisions, especially for the heavy traffic load [4,28]; hence, a lower network throughput is accrued;
- smaller transmission probability results in a lighter traffic load, which increases the packet transmission success rate; however, as Equation (1) indicated, smaller network throughput may be acquired.

Thus, the optimal transmission policy $\omega^{*}$ reflects an optimal trade-off among energy consumption, access delay, and network overall throughput. In order to keep balance between the performance of packet detection and energy consumption, this paper assumes that each EH-ST can transmit $l_{max}$ packet replicas in one frame at most. A specifically admissible policy Ω that indicates the number of transmission packet replicas and transmission probability independent of initial energy state $e_{0}$ is defined below.

**Definition** **1.** *A specifically admissible policy* Ω *is defined as:*

(13) $$\Omega =\left\{\eta \;:\;\left\{\begin{array}{c} \eta \left(0\right)\;=\;0,\;\;\;\;e=0\\ \eta \left(e\right)\;\in \;\left(0,1\right)\;and\;l\;=\;e,\;e<l_{max}\\ \eta \left(e\right)\;\in \;\left(0,1\right]\;and\;l\;=\;l_{max},\;l_{max}\leq e\leq e_{max} \end{array}\right.\right\}.$$

**Remark** **1.** *Note that the set of the specific policy* Ω is made up of two parts. One is the number of transmission packet replicas l and the other one is the probability of transmitting these l packet replicas η(e). In fact, l can be an arbitrary positive integer, and it is no larger than the current energy level e. However, considering the complexity of the analysis procedure and the algorithm design, we predefine a reasonable transmission policy in terms of the number of transmission packet replicas. Specifically, each EH-ST will transmit $l_{max}$ replicas with probability η(e) if $l_{max}\leq e\leq e_{max}$, or it will keep idle with probability 1−η(e). If the current energy level of an EH-ST is less than $l_{max}$, the EH-ST will transmit e replicas with probability η(e). Therefore, we mainly focus on the design of the optimal transmission probability $\eta^{*}$. Since there is a one-to-one mapping between the specific policy Ω *and the transmission probability η, without loss of generality, we refer to $\eta_{u}$ as the policy of EH-ST u in the following parts of this paper.*

Under the policy $\eta \in \Omega^{U}$, the evolution of the energy available in the battery $\left\{E_{k}\right\}$ can be modeled as a Markov chain. For each EH-ST, the state in the chain is defined by $\left\{E_{k}\right\}$, where $E_{k}\in \left\{0,1,2,\ldots ,e_{max}\right\}$ represents the available energy units stored in frame *k*. The transition matrix of the Markov chain is denoted by $P=[p_{mn}]$, where $p_{mn}$ is the probability of one-step transition given by:

(14) $$\mathrm{Pr}\left\{E\left(k+1\right)=e_{n}|E\left(k\right)=e_{m}\right\}.$$

According to the energy harvesting model, energy consumption model, and the defined policy, the transition probability from state $e_{m}$ to state $e_{n}$ is formulated by:

(15) $$p_{mn}=\left\{\begin{array}{c} \beta \left(e_{n},b_{k}\right),\;\;\;\;\;\;\;\;\;\;\;\;\mathrm{if}\;e_{m}=0\;\mathrm{and}\;e_{n}\neq e_{max}\\ 1-\sum_{n=0}^{e_{max}-1}\beta \left(n,b_{k}\right),\;\;\;\;\;\;\;\;\;\;\mathrm{if}\;e_{m}\;=\;0\;\mathrm{and}\;e_{n}\;=\;e_{max}\\ \eta \left(e_{m}\right)\beta \left(e_{n},b_{k}\right),\;\;\;\;\;\;\;\;\;\;\mathrm{if}\;e_{m}\;\neq \;0\;\mathrm{and}\;e_{m}\;\leq \;l_{max}\;\mathrm{and}\;e_{m}\;>\;e_{n}\\ \eta \left(e_{m}\right)\beta \left(e_{n},b_{k}\right)+\left[1-\eta \left(e_{m}\right)\right]\beta \left(e_{n}-e_{m},b_{k}\right),\;\mathrm{if}\;e_{m}\;\neq \;0\;\mathrm{and}\;e_{m}\;\leq \;l_{max}\;\mathrm{and}\;e_{m}\;\leq \;e_{n}\;\mathrm{and}\;e_{n}\;\neq \;e_{max}\\ \eta \left(e_{m}\right)\left[\sum_{n=e_{max}}^{\infty }\;\;\;\beta \left(n,b_{k}\right)\right]+\left[1-\eta \left(e_{m}\right)\right]\left[\sum_{n=e_{n}-e_{m}}^{\infty }\;\;\;\;\beta \left(n,b_{k}\right)\right],\;\mathrm{if}\;e_{m}\;\neq \;0\;and\;e_{m}\;\leq \;l_{max}\;\mathrm{and}\;e_{m}\;=\;e_{max}\\ \eta \left(e_{m}\right)\beta \left(e_{n}-e_{m}+l_{max},b_{k}\right),\;\;\;\;\;\;\;\mathrm{if}\;e_{m}\;\neq \;0\;\mathrm{and}\;e_{m}\;>\;l_{max}\;\mathrm{and}\;e_{m}\;>\;e_{n}\\ \eta \left(e_{m}\right)\beta \left(e_{n}\;-\;e_{m}\;+\;l_{max},b_{k}\right)\;+\;\left[1\;-\;\eta \left(e_{m}\right)\right]\beta \left(e_{n}\;-\;e_{m},b_{k}\right),\;\mathrm{if}\;e_{m}\;\neq \;0\;\mathrm{and}\;e_{m}\;>\;l_{max}\;\mathrm{and}\;e_{m}\;\leq \;e_{n}\;\mathrm{and}\;e_{n}\;\neq \;e_{max}\\ \eta \left(e_{m}\right)\left[\sum_{n=e_{n}\;-\;e_{m}\;+\;l_{max}}^{\infty }\;\;\;\beta \left(n,b_{k}\right)\right]\;+\;\left[1\;-\;\eta \left(e_{m}\right)\right]\left[\sum_{n=e_{n}\;-\;e_{m}}^{\infty }\;\;\;\;\beta \left(n,b_{k}\right)\right],\mathrm{if}\;e_{m}\;\neq \;0\;\mathrm{and}\;e_{m}\;>\;l_{max}\;\mathrm{and}\;e_{m}\;=\;e_{max}\\ 0,\;\;\;\;\;\;\;\;\;\;\;\;\;\;\;\;\mathrm{otherwise} \end{array}\right.$$

which indicates the Markov chain is irreducible under this transmission policy. Therefore, there exists a unique steady-state probability distribution, which is denoted by $\pi_{\eta }\left(e\right),e\in E^{U}$, and it satisfies both $\left(P^{\prime }-I\right)\pi_{\eta }^{\prime }\left(e\right)=0$ and $\sum_{e=0}^{e_{max}}\pi_{\eta }\left(e\right)=1$, where *P* and *I* are transition probability and identity matrices [37]. Thus, the steady-state probability $\pi_{\eta }\left(e\right)$ can be acquired by solving the linear equations. Moreover, the steady-state probability distribution is independent of the initial state $e_{0}$, so Equation (10) can be further deduced as:

(16) Tη(u)=E∑e∈EUπη(e)gηueuUM∏i≠u,jηiei×∏j≠u,i1−ηjej.

Since the packet transmission action $Q_{u,k}$ is based only on the current available energy unit in EH-ST *u*, the energy harvesting process is i.i.d for all EH-STs. Moreover, the energy level of each EH-ST is independent, so $\pi_{\eta }\left(e\right)$ can be represented as:

(17) $$\pi_{\eta }\left(e\right)=\prod_{u}\pi_{\eta_{u}}\left(e_{u}\right),$$

where $\pi_{\eta_{u}}\left(e_{u}\right)$ is the steady-state probability of EH-ST *u* at energy level $e_{u}$. Letting:

(18) $$\begin{array}{cc} G(\eta_{u}) & =\sum_{e=1}^{e_{max}}\pi_{\eta_{u}}\left(e_{u}\right)g\left(\eta_{u}\left(e\right)\right)\\ P(\eta_{u}) & =\sum_{e=1}^{e_{max}}\pi_{\eta_{u}}\left(e_{u}\right)\eta_{u}\left(e\right), \end{array}$$

Equation (16) can be rewritten as:

(19) Tη(u)=EG(ηu)UM∏i≠u,jP(ηi)∏j≠u,i1−P(ηj).

In Equation (19), $G(\eta_{u})$ is the average long-term throughput of EH-ST *u*, assuming there are *M* EH-STs co-transmitting packets in the same frame. $\prod_{i\neq u,j}P\left(\eta_{i}\right)$ is the steady-state probability of other M−1. EH-STs are also transmitting packets, and $\prod_{j\neq u,i}\left(1\;-\;P(\eta_{j})\right)$ represents the steady-state probability of the rest of the U−M EH-STs being in an idle state. According to Equation (11), the network overall throughput under the packet transmission policy η becomes:

(20) Tη=E∑u=1MG(ηu)UM∏i≠u,jP(ηi)∏j≠u,i1−P(ηj).

In order to keep the packet transmission procedure fair, this paper adopts symmetric control policies [28], i.e., all EH-STs employ the same RA policy $\eta_{u}=\eta ,\forall u$. Therefore, Equation (20) can be rewritten as:

(21) Tη=EMG(η)UMP(η)M−11−P(η)U−M.

The total number of EH-STs (*U*) registered in the network can be acquired from the satellite broadcast information. The optimization problem (Equation (12)), under the admissible symmetric policies, can be represented as:

(22) η*=arg maxη∈ΩEMG(η)UMP(η)M−11−P(η)U−M.

## 4. Optimization and Analysis

The optimization problem (Equation (22)) can be separated into two parts. The term M·G(η) represents the expected network total throughput when there are *M* EH-STs co-transmitting packets in the same frame, and the term UMP(η)M−11−P(η)U−M is the probability of *M* EH-STs transmitting packets concurrently in the same frame, which can be regarded as a binomial operator. Moreover, g(η) is concave; thus, Equation (22) is a nonconvex optimization problem. In order to determine the approximated solutions of Equation (22), this paper exploits a game theoretic method which is mentioned in Reference [36] to formulate the random access procedure. Specifically, we use a game theoretic method to model the optimization problem, where each EH-ST *u* acts as a player which optimizes its own transmission policy $\eta_{u}$ to maximize the network throughput (Equation (19)).

This section first analyzes the characteristics of the general Nash equilibrium (NE) of this game. The transmission policy profile is defined as $\eta^{*}=\left(\eta_{1}^{*},\eta_{2}^{*},\ldots ,\eta_{U}^{*}\right)$, which is the joint policy. According to the definition of NE, if any EH-ST *u* adopts the policy $\eta_{u}\neq \eta_{u}^{*}$, while all the other EH-STs use the policy $\eta_{i}^{*}$ (i≠u) to achieve the NE, then a smaller network throughput is obtained. The NE condition must satisfy all the EH-STs, and no player can improve the throughput by deviating unilaterally, i.e.,

(23) $$T_{\eta_{u}^{*},\eta_{-u}^{*}}\geq T_{\eta_{u},\eta_{-u}^{*}},\forall u\in U,\forall \eta_{u}\in \Omega^{U}.$$

By the definition of NE, if an NE (not necessarily symmetric) exists for the game, it must solve ∀u:

(24) ηu*=arg maxηu∈ΩTηu,η−u*=arg maxηu∈ΩE[UM[G(ηu)∏i≠u,jP(ηi*)∏j≠u,i1−P(ηj*)+(1−P(ηu))(∑n≠u,iG(ηn*)∏i≠u,jP(ηi*)∏j≠u,i,n1−P(ηj*)−∑m≠u,jG(ηm*)∏i≠u,j,mP(ηi*)∏j≠u,i1−P(ηj*))]]=arg maxηu∈ΩG(ηu)−P(ηu)∑n≠u,iG(ηn*)1−P(ηj*)−∑m≠u,jG(ηm*)P(ηi*).

In the last step, the optimization formula is divided by the term UM∏i≠u,jP(ηi*)∏j≠u,i1−P(ηj*), and we remove the additive term, which is independent of $\eta_{u}$. Furthermore, we impose the symmetric policy $\eta_{u}^{*}=\eta^{*},\forall u$, and obtain the symmetric NE as follows:

(25) $$\eta^{*}=\underset{\eta \in \Omega }{arg max}G\left(\eta \right)-\Gamma \left(\eta^{*}\right)P\left(\eta \right),$$

where Γ(η) is defined as:

(26) $$\Gamma \left(\eta \right)=G\left(\eta \right)\left(\frac{U-M}{1-P(\eta )}-\frac{M-1}{P(\eta )}\right).$$

$\eta^{*}$ in Equation (25) is the policy that is simultaneously optimal for all the EH-STs, and any single EH-ST unilaterally deviates from the equilibrium condition $\eta^{*}$ yielding a smaller network throughput. G(η) in Equation (25) is the throughput contributed by EH-ST *u* when there are *M* EH-STs accessing the channel simultaneously. The term $\Gamma (\eta^{*})$ can be regarded as a Lagrange operator, which is associated to control the transmission probability of EH-ST *u* and further controls the number of concurrent EH-STs and packet loss rate due to packet collisions. Therefore, the overall objective of Equation (25) is to maximize the individual throughput, so as to maximize the network throughput. Meanwhile, constraints on the average transmission and number of concurrent EH-STs need to be taken into consideration. For a fixed network size *U*, the Lagrange operator decreases with the increase of *M*; however, due to the concavity of g(η), larger *M* will increase the packet loss rate, which is negative to network throughput. Moreover, the larger the throughput or transmission probability of other EH-STs, i.e., $G(\eta^{*})$ or $P(\eta^{*})$, the larger the Lagrange operator $\Gamma (\eta^{*})$; thus, a stringent transmission probability is needed to reduce the packet collisions. The Lagrange operator optimally controls the transmission probability of each EH-ST. Therefore, in order to achieve the maximum throughput of the network, the average transmission probability needs to be properly designed based on the Lagrange operator.

To solve Equation (25), we simplified it to a more general optimization problem, which is represented as follows. For γ≥0:

(27) $$\eta^{\left(\gamma \right)}=\underset{\eta \in \Omega }{arg max}\left[G(\eta )-\gamma P(\eta )\right],$$

and $G\left(\eta \right)-\gamma P\left(\eta \right)=\sum_{e=1}^{e_{max}}\pi_{\eta }\left(e\right)\left[g(\eta (e))-\gamma \eta (e)\right]$. Based on this simplified optimization problem, we can further prove the existence and uniqueness of the symmetric NE in Equation (25), thus finding the solution of Equation (25), which is locally optimal for the original optimization problem (Equation (22)).

Due to the concavity of g(η)[10], $\eta^{\left(\gamma \right)}$ has the following properties.

**Proposition** **1.** *(1) $\eta^{\left(\gamma \right)}$ is uniquely defined, i.e.,*

(28) $$G\left(\eta^{\left(\gamma \right)}\right)-\gamma P\left(\eta^{\left(\gamma \right)}\right)>G\left(\eta \right)-\gamma P\left(\eta \right),\forall \eta \neq \eta^{\left(\gamma \right)};$$

*(2) $\eta^{\left(\gamma \right)}$ is continuous in γ;*

*(3) 0<P(η)≤β(B≥l), $G\left(\eta \right)\geq G\left(\eta_{PLR_{th}}\right)$.*

**Remark** **2.** The first property can be proved by the concavity of g(x); thus, $x_{\gamma }^{*}={arg max}_{x\in [0,1]}g\left(x\right)-\gamma x$ has a unique solution. Due to the continuity of g(x), $\eta^{\left(\gamma \right)}$ is continuous in γ. For the third property, the transmission probability η∈Ω cannot be larger than the energy harvesting rate β(B≥l), where B is number of acquired energy units and l is the number of transmitted packet replicas. Moreover, g(η) is a concave function which is related to PLR, and the relationship between g(η) and PLR is dictated in Equation (9). Since PLR increases with the traffic load increasing, PLR should be below a PLR threshold. Because a large PLR means more packets cannot be detected successfully, which will cause a large delay penalty for the satellite networks, $\eta_{PLR_{th}}$ is the maximum packet transmission probability that ensures the PLR of the network the being below the PLR threshold.

From Equations (25) and (27), we can obtain that $\eta^{*}$ is optimal for Equation (25) if and only if $\eta^{*}=\eta^{\left(\gamma^{*}\right)}$, for $\gamma^{*}>0$, and $\Gamma \left(\eta^{\left(\gamma^{*}\right)}\right)=\gamma^{*}$. In order to prove the existence of the unique solution of Equation (25), we need to prove the following propositions first.

**Proposition** **2.** *$P(\eta^{\left(\gamma \right)})$ is a non-increasing function of γ, and $P(\eta^{\left(0\right)})>0$, $P(\eta^{\left(\infty \right)})=0$.*

**Proof of Proposition** **2.** See Appendix A. ☐

**Proposition** **3.** *$\Gamma (\eta^{\left(\gamma \right)})$ is a non-decreasing, continuous function of γ, and $\Gamma (\eta^{\left(0\right)})>0$, $\Gamma \left(\eta^{\left(\infty \right)}\right)=g^{\prime }\left(0\right)$.*

**Proof of Proposition** **3.** See Appendix B. ☐

Since the proposed policy is a symmetric policy, we further analyze the characteristics of the symmetric NE condition for the game. According to the above propositions, the following theorem proves the existence and uniqueness of the symmetric NE, i.e., the solution of Equation (25).

**Theorem** **1.** There exists a unique solution of the optimization problem (Equation (25)), i.e., $\exists !\eta^{*}\in \Omega$, such that $MG\left(\eta^{*}\right)-\Gamma \left(\eta^{*}\right)P\left(\eta^{*}\right)>MG\left(\eta \right)-\Gamma \left(\eta^{*}\right)P\left(\eta \right),\forall \eta \neq \eta^{*}$. Additionally, the average transmission probability $P\left(\eta^{*}\right)\leq min\left\{\beta \left(B\geq l\right),P\left(\eta_{PLR_{th}}\right)\right\}$.

**Proof of Theorem** **1.** See Appendix C. ☐

Generally, the symmetric NE may be a suboptimal solution of the original optimization problem (Equation (22)), because it is optimal only for the case that EH-ST deviates from the network unilaterally, not symmetrically. Contrarily, if all the EH-STs change the policy by the same quantity (the policy is still symmetric), then it may improve the throughput of the system. Therefore, in this situation, the obtained policy holds for the symmetric NE may not be globally or locally optimal. In the following theorem, we show that the obtained symmetric NE is a locally optimal solution of the optimization problem proposed in this paper.

**Theorem** **2.** *The obtained symmetric NE in Equation (25) is locally optimal for the original optimization problem (Equation (22)).*

**Proof of Theorem** **2.** See Appendix D. ☐

In this paper, we also present an algorithm to determine the optimal policy $\eta^{*}$. From the analysis above, we need to determine the unique $\gamma^{*}$, s.t. $f(\gamma^{*})=0$, where we have previously defined $f\left(\gamma \right)=\Gamma \left(\eta^{\left(\gamma \right)}\right)-\gamma$. Then, according to $\gamma^{*}$, we can obtain the optimal policy $\eta^{*}$ as $\eta^{*}=\eta^{\left(\gamma^{*}\right)}$. Since f(γ) is a continuous decreasing function of γ, f(0)>0, and f(∞)→−∞, we can use the bisection method [38] to search the unique $\gamma^{*}$ which makes $f(\gamma^{*})=0$. Thus, upper bounds $\gamma_{up}$ and lower bounds $\gamma_{low}$ are needed to approach $\gamma^{*}$, where $\gamma_{low}\;<\;\gamma^{*}\;<\;\gamma_{up}$. By computing the value of f(γ) for the updated $\gamma =(\gamma_{up}+\gamma_{low})/2$, the upper and lower bounds are updated and refined recursively until f(γ) satisfies the expected accuracy. In order to compute f(γ), we also need to determine $\Gamma (\eta^{\left(\gamma \right)})$, which can be computed efficiently by a policy iteration algorithm [39]. The lower bound is initialized by $\gamma_{low}=0$. For the upper bound, note that M≈UP(η), and the average throughput gain G(η) is measured by packet/slot which is less than 1. Therefore, the upper bound is initialized by $\gamma_{up}=U$. The algorithm is described in detail in Algorithm 1.

The policy iteration algorithm employed in Algorithm 1 is able to determine the optimal policy $\eta^{\left(\gamma \right)}$ when $\delta_{PIA}\to 0$. The optimality and convergence of the policy iteration algorithm is proven in Reference [39]. Particularly, in the policy improvement step of Algorithm 1, the optimization function has a unique optimal solution, since $d_{\gamma }\left(\eta \left(e\right)\right)$ is a concave function of η(e). The optimal solution can be obtained by taking the derivative of the optimization function w.r.t η(e).

**Remark** **3.** *Algorithm 1 is combined policy iteration algorithm with a bisection method, which can be operated efficiently. For the policy iteration part, we need to calculate the Markov steady state probability distribution $\pi_{\eta^{\left[i\right]}}\left(e\right)$, which can be solved by linear programming using the transition probability (Equation (13)), and its computing complexity is $O(e_{max})$. Furthermore, in order to obtain the value function v(e), we also need to solve the linear system, whose computing complexity is $O(e_{max})$. For the policy improvement step, it needs to take the derivative of the optimization function and then solve the linear function. Thus, the complexity of this step scales as $O(e_{max})$. Finally, these steps are needed to iterated $N_{iter}\left(\delta_{PIA}\right)$ times until it converges. Typically, $N_{iter}\left(\delta_{PIA}\right)$ is no larger than 10 [28]. Therefore, the overall computing complexity of policy iteration algorithm scales as $O(e_{max}N_{iter}\left(\delta_{PIA}\right))$. For the bisection method, the maximum iterations to achieve the expected accuracy $\delta_{BM}$ are $log_{2}\left(U/\delta_{BM}\right)$. Thus, the overall complexity of Algorithm 1 is about $O(e_{max}N_{iter}\left(\delta_{PIA}\right)log_{2}\left(U/\delta_{BM}\right))$.*

**Algorithm 1** (Optimal $\gamma^{*}$ via bisection method )
(1)**Initialization:** Initialize the accuracy of policy iteration algorithm $\delta_{PIA}\;>\;0$ and of bisection method $\delta_{BM}\;>\;0$, $\gamma_{low}^{\left[i\right]}\;=\;0$ and $\gamma_{up}^{\left[i\right]}\;=\;U$, $\eta^{\left[j\right]}\left(e\right)\;\in \;\left(0,min\left\{\beta \left(B\geq l\right),\eta_{PLR_{th}}\right\}\right)$; i=j=0;(2)**Policy Optimization:** Let $\gamma^{\left[i\right]}=\left(\gamma_{up}^{\left[i\right]}+\gamma_{low}^{\left[i\right]}\right)/2$ and determine $\eta^{\left(\gamma \right)}$ by the following policy iteration steps:**Policy Evaluation:** Calculate the value function $v_{\eta }\left(e\right)$ for e>0, where $v_{\eta }\left(e\right)$ is the solution of the linear system $v_{\eta^{\left[j\right]}}\left(e\right)\;-\;\sum_{\varepsilon \;\in \;E}P_{\eta^{\left[j\right]}}\left(\varepsilon \;|\;e\right)\;-\;v_{\eta^{\left[j\right]}}\left(\varepsilon \right)\;=\;d_{\gamma^{\left[i\right]}}\left(\eta^{\left[j\right]}\left(e\right)\right)\;-\;D\gamma^{\left[i\right]}\left(\eta^{\left[j\right]}\right),e\;\in \;E$, where $D_{\gamma }\left(\eta \right)\;=\;MG\left(\eta \right)\;-\;\gamma P\left(\eta \right)$, $d_{\gamma }\left(\eta \right)\;=\;U\eta \left(e\right)g\left(\eta \left(e\right)\right)\;-\;\gamma \eta \left(e\right)$ and $P_{\eta^{\left[j\right]}}\left(\varepsilon \;|\;e\right)$ is the Markov transition probability from energy level *e* to ε.**Policy Improvement:** Determine the new policy by solving the following optimization problem $\eta^{\left[j+1\right]}\left(e\right)$ = ${arg max}_{\eta \;\in \;\left(0,min\left\{\beta \left(B\geq l\right),\eta_{PLR_{th}}\right\}\right)}\;d_{\gamma^{\left[i\right]}}\left(\eta^{\left[j\right]}\left(e\right)\right)\;+P_{\eta^{\left[j\right]}}\left(\varepsilon \;|\;e\right)\;$. If $\eta^{\left[j+1\right]}\left(e\right)\;>\;min\left\{\beta \left(B\geq l\right),\eta_{PLR_{th}}\right\}$, then $\eta^{\left[j+1\right]}\left(e\right)\;=\;min\left\{\beta \left(B\geq l\right),\eta_{PLR_{th}}\right\}$.**Termination Test for Policy Iteration Algorithm:** If $\left|D\gamma^{\left[i\right]}\left(\eta^{\left[j\right]}\right)-D\gamma^{\left[i\right]}\left(\eta^{\left[j+1\right]}\right)\right|<\delta_{PIA}$, $\eta^{\left[j+1\right]}$ is the optimal policy and $\eta^{\left(\gamma^{\left[i\right]}\right)}=\eta^{\left[j+1\right]}$. Otherwise, i:=i+1, and repeat from the policy evaluation step.(3)**Calculation $f(\gamma^{\left[i\right]})$:** Calculate $f\left(\gamma^{\left[i\right]}\right)=\Gamma \left(\eta^{\left(\gamma^{\left[i\right]}\right)}\right)-\gamma^{\left[i\right]}$ under the policy $\eta^{\left(\gamma^{\left[i\right]}\right)}$.(4)
**Termination Test for Bisection Method:**
If $\left|f(\gamma^{\left[i\right]})\right|\;<\;\delta_{BM}$, $\eta^{*}\;=\;\eta^{\left(\gamma^{\left[i\right]}\right)}$;If $f\left(\gamma^{\left[i\right]}\right)<-\delta_{BM}$, update the bounds $\gamma_{up}^{\left[i+1\right]}:=\gamma^{\left[i\right]}$ and $\gamma_{low}^{\left[i+1\right]}:=max\left\{\gamma_{low}^{\left[i\right]},\Gamma \left(\eta^{\left(\gamma^{\left[i\right]}\right)}\right)\right\}$ respectively, repeat from step (2) and update the counter i:=i+1;If $f\left(\gamma^{\left[i\right]}\right)>\delta_{BM}$, update the bounds $\gamma_{low}^{\left[i+1\right]}:=\gamma^{\left[i\right]}$ and $\gamma_{up}^{\left[i+1\right]}:=min\left\{\gamma_{max}^{\left[i\right]},\Gamma \left(\eta^{\left(\gamma^{\left[i\right]}\right)}\right)\right\}$ respectively, repeat from step (2) and update the counter i:=i+1.



## 5. Simulation Results

This section provides some simulation results to evaluate the proposed random access policy. It is assumed that the number of time slots in a frame *N* is 200 and each packet occupies one time slot. The length of each packet is 100bits and the data are modulated by QPSK. The received energy per symbol noise power spectral density ratio $E_{s}/N_{0}$ of each packet is assumed to be equal with 10 dB. Additive white Gaussian noise (AWGN) is added before CRDSA demodulating, and the maximum decoding iteration is to set $N_{iter}^{max}=15$. For CRDSA, a packet is either clear (not collided with other packets) or interfered with other packets entirely [4]. For the clear packets, they can be decoded easily even without FEC, since $E_{s}/N_{0}$ is large enough to decode the packets correctly. For the entirely collided packets, they can hardly be decoded correctly even with FEC, since the power of the overlapped packets is nearly the same [11]. Thus, we do not consider FEC in this paper. Actually, a power unbalance scenario and proper transmission power selection schemes combined with FEC can further boost the performance of the CRDSA [40], but the analytical model needs to be redesigned, which is out of the scope of this paper. This paper aims to provide a common but effective train of thought of designing a distributed RA policy for energy harvesting devices in satellite communication networks. Thus, the application of FEC is left for future research together with the power unbalance scenario and some variant CRDSA protocols. The main simulation parameters in this section are listed in Table 1.

**Figure 3:**
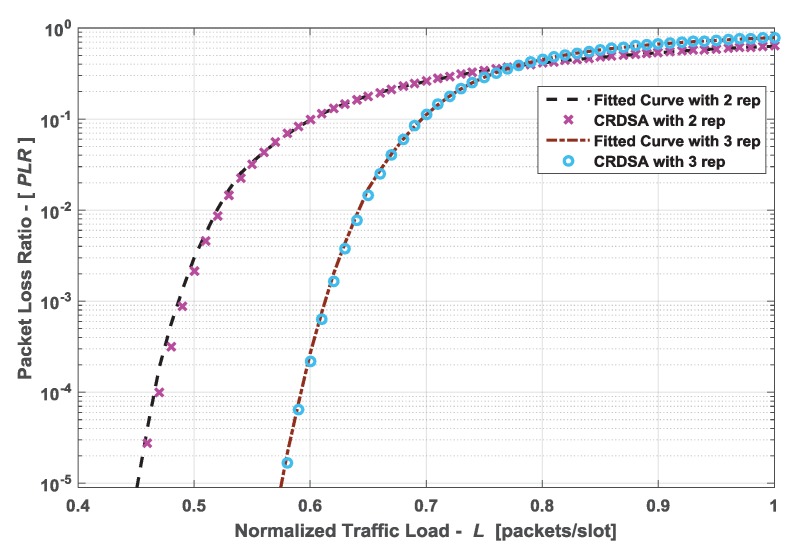
Packet loss ratio PLR curves comparison of the fitted curves and CRDSA with 2 and 3 replicas, number of time slots N=200, QPSK modulation, $E_{s}/N_{0}=10$ dB.

Since CRDSA adopts the scheme of successive interference cancellation, the value of PLR is obtained by an iteration method for a specific traffic load [4,10]. Therefore, the expression of PLR(L) cannot be obtained directly. This paper uses the method of curve fitting to approximate the PLR function. In order to guarantee the PLR(L) increasing and g(η) concave in the range (0,1), we adopt a Gaussian model with 8 terms to fit the curve, and the simulation results are shown in Figure 3. PLR value of CRDSA is computed by the method mentioned in Reference [10]. The traffic load is normalized by M/N, where *M* is the number of concurrent terminals. Figure 3 shows the fitted curves are well matched with CRDSA PLR curves for both 2 and 3 replicas.

### 5.1. Throughput and PLR

**Figure 4:**
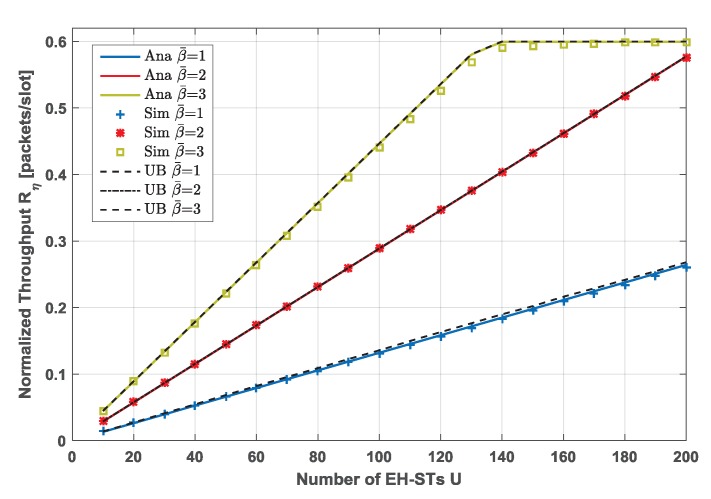
Normalized network throughput for different network sizes, under different EH rates $\bar{\beta }\in \left\{1,2,3\right\}$, $e_{max}=10$. For random access (RA) schemes, number of time slots in each frame N=200, QPSK modulation, $E_{s}/N_{0}=10$ dB.

Figure 4 illustrates the network throughput of the proposed policy obtained by simulation and theoretical analysis. We plot the relationship between the normalized network throughput and the number of EH-STs in the network *U*, and consider different scenarios by varying the energy harvesting rate $\bar{\beta }\in \left\{1,2,3\right\}$. The normalized throughput is defined as T(L)=(1−PLR(L))L, where the traffic load *L* is normalized by M/N. The capacity of the battery is assumed to be $e_{max}=10$ and the PLR threshold is set $10^{-3}$. The simulation results indicate that the analytical network throughput in different EH rate scenarios is well matched with that simulated in the same scenario. For the lower EH rates $\bar{\beta }=1$ and $\bar{\beta }=2$, the network throughput increases with the number of EH-STs in the network linearly, because in these scenarios, the transmission probability is constrained by the EH rate. Moreover, under a lower EH rate, although the network size increases, the average number of concurrent EH-STs is still less than what CRDSA can support, i.e., the achieved PLR does not exceed the threshold. Due to the properties of g(η), the network throughput increases with the network size for the lower EH rates. As the EH rate grows, each EH-ST has more transmission opportunities. Since the CRDSA protocol has a good performance on recovering collided packets under moderate traffic load, the network will gain higher throughput under a higher EH rate. When the EH rate is high enough ($\bar{\beta }=3$), the throughput of the network increases with the network size first, but afterwards it tends to be flat. This is because when the network size becomes larger, more EH-STs have enough energy to transmit the packet. For the larger network size, packet collisions become the bottleneck of the performance. The policy will control the transmission probability of each EH-ST to guarantee the PLR is under the threshold.

In addition, we also plot the upper bounds (UB) of the network throughput for different scenarios, which are represented by the black dashed line in Figure 4. Since g(η) is a concave function, G(η)≤g(P(η)) from Jensen’s inequality [41]. Moreover, $P\left(\eta \right)\leq min\left\{\beta \left(B\geq l\right),P\left(\eta_{PLR_{th}}\right)\right\}$; thus, the upper bound of the network throughput can be obtained as:

(29) Tη≤arg maxx∈0,minβ(B≥l),P(ηPLRth)EMg(x)UMxM−1(1−x)U−M.

We notice that the proposed policy calculated by Algorithm 1 closely approaches the upper bound under each scenario and the performance degradation for each case is within 5% w.r.t the upper bound. This indicates the locally optimal solution achieved by the symmetric NE is a near-global optimum.

**Figure 5:**
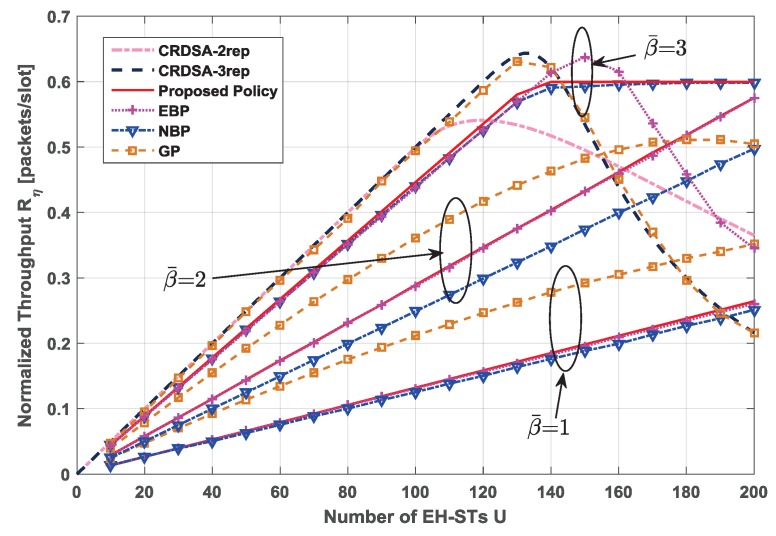
Normalized throughput of different policies comparison for different network sizes, under different EH rates $\bar{\beta }\in \left\{1,2,3\right\}$, $e_{max}=10$. For RA schemes, number of time slots in each frame N=200, QPSK modulation, $E_{s}/N_{0}=10$ dB.

**Figure 6:**
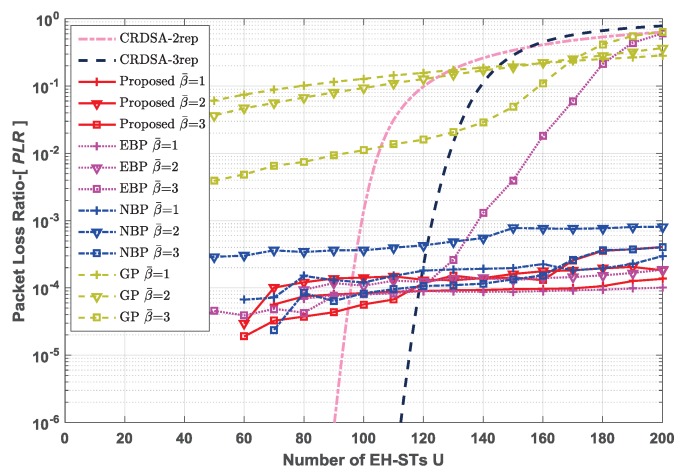
Comparison of packet loss ratio of different policies for different network sizes, under different EH rates $\bar{\beta }\in \left\{1,2,3\right\}$, $e_{max}=10$. For RA schemes, number of time slots in each frame N=200, QPSK modulation, $E_{s}/N_{0}=10$ dB.

In addition to the proposed policy, we also evaluated the performance of the following policies, which are based on the baseline mentioned in Equation (12): The energy-balanced policy (EBP), where each EH-ST transmits the packets with the probability of β(B≥l); the network-balanced policy (NBP), where each EH-ST transmits the packets with the probability of $\eta_{PLR_{th}}$ so as to maximize the throughput of the network; and the greedy policy (GP), where each EH-ST transmits the packets with the probability of 1 as long as it has enough energy. The simulation results for throughput and packet loss ratio are represented in Figure 5 and Figure 6, respectively. In addition, we also present the performance of CRDSA (without consideration of the EH process and the limitation of energy) with 2 and 3 replicas to compare with other policies.

For the lower EH rates $\bar{\beta }=1$ and $\bar{\beta }=2$ cases, the normalized throughput of the proposed policy, EBP and NBP all increase linearly with the increase of network size. The performance of EBP is nearly the same as that of the proposed policy, because in these scenarios, the transmission probability of each EH-ST is limited by the EH rate. The performance of NBP is a little worse than that of the proposed policy and EBP, because in these scenarios, $\eta_{PLR_{th}}>\beta \left(B\geq l\right)$, which means the transmission probability of each EH-ST is larger than the energy arrival rate. Therefore, more EH-STs are in the state of energy exhausted. From the perspective of PLR, all these three policies are able to control the PLR under the PLR threshold in the lower EH rate cases. Interestingly, the performance of GP seems better than the other three policies. In fact, under GP, EH-STs will always transmit packets as long as they have energy, which increases the traffic load; thus, GP can achieve a higher throughput. However, due to the low EH rate, most EH-STs have no energy to transmit packet replicas, which makes the collided packets unable to be recovered and increases the PLR. Actually, Figure 6 shows the PLR of GP is beyond the threshold even though the traffic load is light. A large PLR leads to packets retransmission frequent, which will cause unacceptable large communication latencies in the satellite networks and expand more energy units for the EH-STs.

On the other hand, when $\bar{\beta }=3$, the performance of NBP and EBP is close to the proposed policy for the light and moderate traffic load. When the traffic load becomes heavier, the normalized throughput of NBP and the proposed policy tends to be flat at 0.6 packets/slot, while the performance of EBP drops dramatically. This is because for the lighter traffic load, $\beta \left(B\geq l\right)<\eta_{PLR_{th}}$, the performance of these three policies is still constrained by the EH rate. As the network size grows larger, packet collisions become severe. EH-STs employ NBP and the proposed policy transmitting the packets with probability of $\eta_{PLR_{th}}$, but those who employ EBP transmit the packets with a probability of β(B≥l). At this time, $\beta \left(B\geq l\right)>\eta_{PLR_{th}}$, which results in the PLR of EBP, exceeds the threshold. Thus, the performance of EBP becomes worse for the larger network size. GP seems to behave better than the other three policies in the small and medium network size scenario. However, as mentioned above, the PLR of GP exceeds the threshold even for the light traffic load. Moreover, the maximum normalized throughput of EBP and GP is similar with that of CRDSA with 3 replicas, which almost reaches 0.64 packets/slot. However, when the performance achieves the peak, the PLR is beyond the threshold. Notice that all the policies cannot perform as well as the CRDSA protocols for the small and medium network size, because conventional CRDSA does not consider the limitation of energy and the EH process. Therefore, terminals employing conventional CRDSA protocols are assumed to always have enough energy to transmit their packet replicas. In other words, if the EH rate is high enough, the proposed RA scheme can perform as well as CRDSA protocols.

### 5.2. Data Delivery Probability

**Figure 7:**
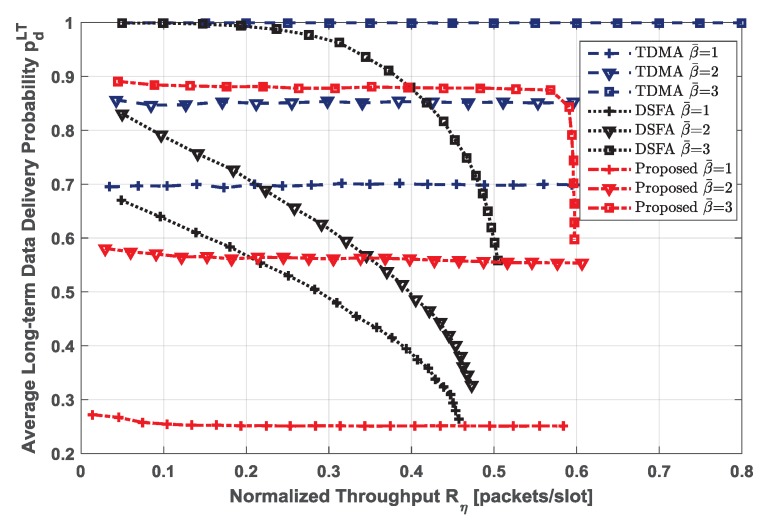
Trade-off between average long-term data delivery probability and network normalized throughput under different EH rates $\bar{\beta }\in \left\{1,2,3\right\}$, $e_{max}=10$. For access schemes, number of time slots in each frame N=200 (only for time division multiple access (TDMA) and the proposed scheme, since the frame length of dynamic frame slotted Aloha (DFSA) is dynamic), QPSK modulation, $E_{s}/N_{0}=10$ dB.

The trade-off between the average long-term data delivery probability and the network normalized throughput is shown in Figure 7 under different EH rates $\bar{\beta }\in \left\{1,2,3\right\}$. System parameters are the same with the previous simulations. We evaluated the performance of two centralized access protocols, which are time division multiple access (TDMA) and dynamic frame slotted Aloha (DFSA) [20,21], to compare with that of the proposed distributed RA scheme. TDMA and DFSA are centralized access protocols, while the proposed policy is based on a random access scheme, which is different from the other two protocols in terms of the nature of mechanisms. In fact, the centralized schemes are expected to achieve higher throughput performance than the RA schemes, because packet collisions can be avoided by proper management at the central controller. However, in the energy-constrained networks, the centralized access schemes cannot always achieve such high data delivery probability and throughput due to energy shortage. In the simulations, we compare the performance of these three schemes from the perspective of energy constraint, which seems reasonable. The packet delivery probability of all these three schemes increases with the increase of $\bar{\beta }$. TDMA always outperforms DFSA and the proposed RA scheme in terms of data delivery probability. Since time slots have been preallocated to each EH-ST, it does not suffer packet collisions. Therefore, the data delivery probability of TDMA is determined by the EH rate. DSFA can adjust the length of frame dynamically according to the number of active EH-STs, and it offers retransmission opportunities for the EH-STs. For the lower EH rates, many EH-STs are in a state of energy shortage; thus, the data delivery probability of DSFA is limited by the EH rate. Moreover, a larger frame makes more EH-STs deliver their data to the satellite successfully, but causes lower throughput. Unlike DSFA, the proposed distributed RA scheme balances well for both data delivery probability and throughput. This is because the delivery probability of the proposed scheme is restricted not only by $\bar{\beta }$, but also by $\eta_{PLR_{th}}$. Thanks to the interference cancellation mechanism, the maximum throughput of the proposed RA scheme is higher than that of DFSA. However, transmitting more packet replicas will consume more energy. Compared with TDMA and DFSA (they only transmit one physical packet once), more EH-STs do not have enough energy to deliver the data. That is why the data delivery probability of the proposed RA schemes is only about 0.26 when $\bar{\beta }=1$. For the lower EH rates (e.g., $\bar{\beta }=1$ and $\bar{\beta }=2$), the EH rate dominates the delivery probability. When EH rates become higher, almost every EH-ST has enough energy to send data. At this time, the proposed policy needs to guarantee the PLR being not beyond the PLR threshold; thus, $\eta_{PLR_{th}}$ determines the delivery probability. Specifically, when $\bar{\beta }=3$, the delivery probability of the proposed scheme presents to be flat for the lower and moderate throughput but drops dramatically to control the PLR for the higher throughput.

Notice that both TDMA and DFSA are centralized access protocols, and the central controller will allocate the time slots or adjust the frame length to improve the performance. However, the proposed distributed RA scheme can achieve acceptable throughput under the higher data delivery probability, which even performs better than DFSA. In addition, the proposed distributed RA scheme does not need the procedure of resource allocation and controls the PLR strictly, which reduces the communication delays caused by resource assignment and packet retransmission. Therefore, the proposed distributed RA scheme is more suitable for the future low-cost interactive satellite communication scenario with a large network size and high EH rate.

### 5.3. Packet Delay Assessment

**Figure 8:**
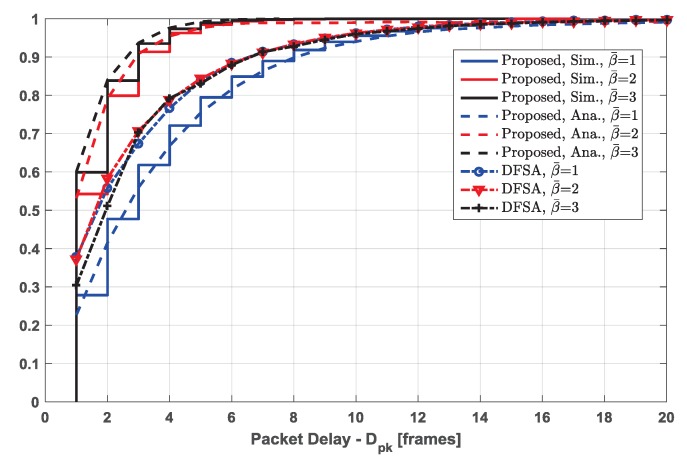
Packet delay cumulative distribution for the proposed scheme and DFSA with number of EH-STs U=200 under different EH rates $\bar{\beta }\in \left\{1,2,3\right\}$, $e_{max}=10$. For access schemes, number of time slots in each frame N=200 (only for the proposed scheme. For DFSA, the packet delay in frames is normalized, since the frame length of DFSA is dynamic), QPSK modulation, $E_{s}/N_{0}=10$ dB.

The metric of packet delay is assessed for the proposed scheme and DFSA protocol in a heavy traffic load, and the results are shown in Figure 8. For the proposed scheme, we evaluate the performance of packet delay via both simulation (solid lines) and analytical method (dash lines), and they are well matched under different energy harvesting rates. Moreover, as the energy harvesting rate increases, the performance of packet delay becomes better. For instance, the probabilities of a packet being received successfully within 2 frames are about 0.48, 0.8, and 0.85 under the EH rates $\bar{\beta }=1,2$, and 3 respectively. This is because the (re)transmission probability is not only constrained by the current energy level, but also the PLR threshold, regardless of the packet being transmitted for the first time or being retransmitted. Therefore, with the constraint of PLR threshold, almost all the packets can be correctly received as long as they are transmitted, and retransmissions will not block the channel. According to the analysis in Section 5.2, for the lower EH rates (e.g., $\bar{\beta }=1$ and $\bar{\beta }=2$), the EH rate dominates the delivery probability, while for the higher EH rate, $\eta_{PLR_{th}}$ determines the delivery probability. Thus, the packet delay when $\bar{\beta }=3$ performs slightly better than when $\bar{\beta }=2$.

Since TDMA is a collision-free protocol, no retransmissions occur in this system. Therefore, we only adopt DFSA to compare with the proposed scheme in terms of packet delay. For DFSA, packets are transmitted as long as EH-STs have energy, and no replicas are used, which will consume less energy. For different EH rates $\bar{\beta }\in \left\{1,2,3\right\}$, almost every EH-ST has energy to transmit its packet; thus, their performances of packet delay are nearly the same. Since each packet is only transmitted once in a frame, EH-STs applying DFSA have more energy and chances to retransmit their data when the EH rate is low ($\bar{\beta }=1$) compared with those applying the proposed scheme. Therefore, the packet delay performance of DFSA is better than that of the proposed scheme when $\bar{\beta }=1$. However, without a packet collision resolution mechanism, EH-STs applying DFSA have to retransmit their data more times. Therefore, when the EH rate becomes higher, EH-STs applying the proposed scheme have more opportunities to transmit their data, and the packet delay performance of the proposed scheme is superior to that of DFSA.

## 6. Conclusions

This paper considers a satellite communication network made up by a GEO satellite and multiple EH-STs. The EH-STs transmit data packets to the satellite randomly over a shared wireless collision channel. Packet replicas and a successive interference cancellation mechanism are adopted to improve the network throughput. Considering the EH process and energy constraints of EH-STs, we have designed a distributed RA scheme aiming to maximize the average long-term network throughput. Firstly, we developed an analytical model of the average long-term throughput with constraints of packet loss ratio and energy and adopted a game theoretic method to approximate the solution of the nonconvex optimization problem. Then, we characterized the symmetric NE of the game and proved its existence and uniqueness, which indicated it is a locally optimal solution of the original optimization problem. A policy iteration algorithm combined with a bisection method was used to compute the symmetric NE. Finally, we evaluated the proposed RA scheme by simulations. Simulation results showed that the proposed RA scheme is more suitable for the large network size and high EH rate scenario, which is applicable to EH devices in the future low-cost interactive satellite communication system.

This paper provides a common but effective train of thought of designing a distributed RA policy for energy harvesting devices in satellite communication networks, but it is based on a simplified version of CRDSA. In order to further improve the policy, we will concentrate on researching the following works in the future. First, we will extent the policy to an asynchronous version, since it is more suitable to the energy constrained system (time synchronization will cost extra signalings and energy). Second, we will investigate the performance of using irregular replicas based on the analysis method in this paper. In addition to the number of packet replicas, the effect of power imbalance and transmission power selection are also important factors that need to be taken into consideration in future work.

## Figures and Tables

**Table 1:** Simulation parameters. EH-ST: Energy harvesting satellite terminal.

Parameters	Settings
Number of time slots in a frame	*N* = 200
Length of each packet	100 bits
Modulation scheme	QPSK
Energy per symbol noise power spectral density ratio	$E_{s}/N_{0}$ = 10 dB
Maximum decoding iteration	$N_{iter}^{max}$ = 15
Number of EH-STs	*U* = [0:20:200]
Number of replicas in CRDSA	l≤3
Energy harvesting rates	$\bar{\beta }\in \left\{1,2,3\right\}$
Battery capacity	$e_{max}$ = 10

## Appendix A

**Proof of Proposition** **2.** We prove this proposition by contradiction. Assume $\gamma_{1}>\gamma_{2}$, and $P\left(\eta^{\left(\gamma_{1}\right)}\right)>P\left(\eta^{\left(\gamma_{2}\right)}\right)$. Then, according to the first property of Proposition 1 and the hypothesis, we have:

(A1) $$\begin{array}{c} G\left(\eta^{\left(\gamma_{2}\right)}\right)\;\;-\;\;\gamma_{2}P\left(\eta^{\left(\gamma_{2}\right)}\right)\;\;\geq \;\;G\left(\eta^{\left(\gamma_{1}\right)}\right)\;\;-\;\;\gamma_{1}P\left(\eta^{\left(\gamma_{1}\right)}\right)\;\;+\;\;\left(\gamma_{1}\;\;-\;\;\gamma_{2}\right)P\left(\eta^{\left(\gamma_{1}\right)}\right)\\ >G\left(\eta^{\left(\gamma_{1}\right)}\right)-\gamma_{1}P\left(\eta^{\left(\gamma_{1}\right)}\right)+\left(\gamma_{1}-\gamma_{2}\right)P\left(\eta^{\left(\gamma_{2}\right)}\right)\\ \geq G\left(\eta^{\left(\gamma_{2}\right)}\right)-\gamma_{1}P\left(\eta^{\left(\gamma_{2}\right)}\right)+\left(\gamma_{1}-\gamma_{2}\right)P\left(\eta^{\left(\gamma_{2}\right)}\right)\\ =G\left(\eta^{\left(\gamma_{2}\right)}\right)-\gamma_{2}P\left(\eta^{\left(\gamma_{2}\right)}\right). \end{array}$$

Hence, we obtain a contradictory result. For the second part, when γ=0, the policy $\eta^{\left(0\right)}$ is not an idle policy. Thus, $P(\eta^{\left(0\right)})$. When γ→∞, in order to maximize the optimization problem (Equation (26)), $P(\eta^{\left(\infty \right)})\to 0$. Thus, the proposition is proved. ☐

## Appendix B

**Proof of Proposition** **3.** The continuity of the function follows the first property of Proposition 1. When γ=0, we have:

(A2) $$\Gamma \left(\eta^{\left(0\right)}\right)\;=\;G\left(\eta^{\left(0\right)}\right)\;\;\left(\frac{U-M}{1-P\left(\eta^{\left(0\right)}\right)}\;-\;\frac{M-1}{P\left(\eta^{\left(0\right)}\right)}\right)\;\in \;\left(0,1\right).$$

Since G(η) means the average throughput, $0<G\left(\eta^{\left(0\right)}\right)\leq 1$. From Propositions 1 and 2, we know that $0<P\left(\eta^{\left(0\right)}\right)\leq \beta \left(B\geq l\right)$ and M≤U. Therefore, $\Gamma \left(\eta^{\left(0\right)}\right)$ is positive and bounded. On the other hand, when γ→∞, $P\left(\eta^{\left(\infty \right)}\right)\to 0$ and $G\left(\eta^{\left(\infty \right)}\right)\to 0$. Since the expected number of co-transmitting EH-STs *M* can be approximated by $\eta^{\left(\gamma \right)}U$, $\Gamma \left(\eta^{\left(\gamma \right)}\right)$ can be simplified as $G\left(\eta^{\left(\gamma \right)}\right)/P\left(\eta^{\left(\gamma \right)}\right)$. By L’Hospital’s rule, $\Gamma \left(\eta^{\left(\infty \right)}\right)=g^{\prime }\left(0\right)$. Then, we prove $\Gamma \left(\eta^{\left(\gamma \right)}\right)$ is a nondecreasing function of γ, i.e., $\Gamma \left(\eta^{\left(\gamma_{1}\right)}\right)\geq \Gamma \left(\eta^{\left(\gamma_{2}\right)}\right)$ for $\gamma_{1}>\gamma_{2}\geq 0$. Since $\Gamma \left(\eta^{\left(\gamma \right)}\right)$ can be simplified as $G\left(\eta^{\left(\gamma \right)}\right)/P\left(\eta^{\left(\gamma \right)}\right)$, we only need to prove $G\left(\eta^{\left(\gamma_{2}\right)}\right)P\left(\eta^{\left(\gamma_{1}\right)}\right)-G\left(\eta^{\left(\gamma_{1}\right)}\right)P\left(\eta^{\left(\gamma_{2}\right)}\right)\leq 0$ which can be represented as:

(A3) $$\begin{array}{c} \left[G\left(\eta^{\left(\gamma_{2}\right)}\right)\;-\;\gamma_{2}P\left(\eta^{\left(\gamma_{2}\right)}\right)\right]P\left(\eta^{\left(\gamma_{1}\right)}\right)\;-\;\left[G\left(\eta^{\left(\gamma_{1}\right)}\right)\;-\;\gamma_{1}P\left(\eta^{\left(\gamma_{1}\right)}\right)\right]\\ \times P\left(\eta^{\left(\gamma_{2}\right)}\right)+\;\gamma_{2}P\left(\eta^{\left(\gamma_{2}\right)}\right)P\left(\eta^{\left(\gamma_{1}\right)}\right)-\gamma_{1}P\left(\eta^{\left(\gamma_{1}\right)}\right)P\left(\eta^{\left(\gamma_{2}\right)}\right)\leq 0. \end{array}$$

According to the fact that $\eta^{\left(\gamma_{2}\right)}$ is optimal for Equation (27), when $\gamma =\gamma_{2}$. Therefore, $G\left(\eta^{\left(\gamma_{2}\right)}\right)\;-\;\gamma_{2}P\left(\eta^{\left(\gamma_{2}\right)}\right)\geq G\left(\eta^{\left(\gamma_{1}\right)}\right)\;-\;\gamma_{2}P\left(\eta^{\left(\gamma_{1}\right)}\right)$. A sufficient condition that holds for Equation (A3) is that $\left[G\left(\eta^{\left(\gamma_{2}\right)}\right)\;-\;\gamma_{2}P\left(\eta^{\left(\gamma_{2}\right)}\right)\right]P\left(\eta^{\left(\gamma_{1}\right)}\right)\;-\;\left[G\left(\eta^{\left(\gamma_{2}\right)}\right)\;-\;\gamma_{1}P\left(\eta^{\left(\gamma_{2}\right)}\right)\right]\times P\left(\eta^{\left(\gamma_{2}\right)}\right)+\;\gamma_{2}P\left(\eta^{\left(\gamma_{2}\right)}\right)P\left(\eta^{\left(\gamma_{1}\right)}\right)-\gamma_{1}P\left(\eta^{\left(\gamma_{1}\right)}\right)P\left(\eta^{\left(\gamma_{2}\right)}\right)\leq 0$, which is equivalent to $\left[G\left(\eta^{\left(\gamma_{2}\right)}\right)\;-\;\gamma_{2}P\left(\eta^{\left(\gamma_{2}\right)}\right)\right]\left[P\left(\eta^{\left(\gamma_{1}\right)}\right)\;-P\left(\eta^{\left(\gamma_{2}\right)}\right)\right]\leq 0.$ It is obviously true for $P\left(\eta^{\left(\gamma_{2}\right)}\right)\;\geq P\left(\eta^{\left(\gamma_{1}\right)}\right)$, which has been proven in Proposition 2. Thus, this proposition is proven. ☐

## Appendix C

**Proof of Theorem** **1.** The existence and uniqueness of the solution of Equation (25) can be proven by Proposition 3. We define $f\left(\gamma \right)≜\Gamma \left(\eta^{\left(\gamma \right)}\right)-\gamma$. Based on Proposition 3, f(γ) has the properties of $f\left(\gamma \right)=\Gamma \left(\eta^{\left(0\right)}\right)>0$ and $\lim_{\gamma \to \infty }f\left(\gamma \right)=g^{\prime }\left(0\right)-\infty$. Since g(η) is concave, and $g^{\prime }\left(0\right)$ is a limited positive number [10], $\lim_{\gamma \to \infty }f\left(\gamma \right)=-\infty$ and there exists a unique $\gamma^{*}\in \left(0,\infty \right)$ satisfying $f(\gamma^{*})=0$, i.e., $\Gamma \left(\eta^{\left(\gamma^{*}\right)}\right)=\gamma^{*}$, which guarantees $\eta^{\left(\gamma^{*}\right)}$ is optimal for Equation (24). Then, we explain the second part of the theorem that $P\left(\eta^{\left(\gamma^{*}\right)}\right)\leq min\left\{\beta \left(B\geq l\right),P\left(\eta_{PLR_{th}}\right)\right\}$. From the third property of P1, the average transmission probability should not be larger than the energy harvesting rate, i.e., $P\left(\eta^{\left(\gamma^{*}\right)}\right)\leq \beta \left(B\geq l\right)$. Moreover, according to the nature of the MAC protocol CRDSA, as the traffic load increases, PLR increases as well. On the other hand, the throughput of the network increases with the increase of traffic load first but decreases sharply when the traffic load is larger than a threshold value [4]. The traffic load is directly related with the transmission probability and the raffic load threshold value mentioned above corresponds to a PLR threshold value. In order to maximize the network throughput, the average transmission probability should not be larger than the transmission probability that makes the network PLR larger than the PLR threshold, i.e., $P\left(\eta^{\left(\gamma^{*}\right)}\right)\leq P\left(\eta_{PLR_{th}}\right)$, where $\eta_{PLR_{th}}$ is the policy that achieves the PLR threshold. Thus, the theorem is proven. ☐

## Appendix D

**Proof of Theorem** **2.** As mentioned above, the obtained policy $\eta^{*}$ is a global optimum for Equation (25); thus, the gradient of the objective function in Equation (25) w.r.t η (denoted as $\Delta_{\eta }\left(\cdot \right)$) is zero when $\eta =\eta^{*}$. Moreover, its Hessian matrix w.r.t η (denoted as $H_{\eta }\left(\cdot \right)$) is semidefinite negative when $\eta =\eta^{*}$ [36]. Therefore, for the symmetric policy $\eta^{*}$, we have:

(A4) $${\left[\Delta_{\eta }\left(G(\eta )\right)-\Gamma \left(\eta^{*}\right)\Delta_{\eta }\left(P(\eta )\right)\right]}_{\eta =\eta^{*}}=0,$$

(A5) $${\left[H_{\eta }\left(G(\eta )\right)-\Gamma \left(\eta^{*}\right)H_{\eta }\left(P(\eta )\right)\right]}_{\eta =\eta^{*}}⪯0.$$

The gradient of the original optimization problem (Equation (22)) is derived as:

(A6) Δη(Rη)=E{UMM[P(η)M−1(1−P(η))U−MΔη(G(η))+(M−1)G(η)(1−P(η))U−MP(η)M−2Δη(P(η))−(U−M)G(η)(1−P(η))U−M−1P(η)M−1Δη(P(η))]}=EUMMΔη(G(η))−G(η)U−M1−P(η)−M−1P(η)Δη(P(η)).

When $\eta =\eta^{*}$, substituting Equation (A4) in Equation (A6), we can obtain that ${\left[\Delta_{\eta }\left(R_{\eta }\right)\right]}_{\eta =\eta^{*}}=0$. On the other hand, by further deriving Equation (A5), the Hessian matrix can be derived as Equation (A7).

(A7) Hη(Rη)=UMM[P(η)M−1(1−P(η))U−MHη(G(η))+(M−1)P(η)M−2(1−P(η))U−MΔη(G(η))Δη(P(η))T−(U−M)P(η)M−1(1−P(η))U−M−1Δη(G(η))Δη(P(η))T+(M−1)G(η)P(η)M−2(1−P(η))U−MHη(P(η))+(M−1)P(η)M−2(1−P(η))U−MΔη(P(η))Δη(G(η))T+(M−1)(M−2)G(η)P(η)M−3(1−P(η))U−MΔη(P(η))Δη(P(η))T−(M−1)(U−M)G(η)P(η)M−2(1−P(η))U−M−1Δη(P(η))Δη(P(η))T−(U−M)G(η)P(η)M−1(1−P(η))U−M−1Hη(P(η))−(U−M)P(η)M−1(1−P(η))U−M−1Δη(P(η))Δη(G(η))T−(M−1)(U−M)G(η)P(η)M−2(1−P(η))U−M−1Δη(P(η))Δη(P(η))T+(U−M)(U−M−1)G(η)P(η)M−1(1−P(η))U−M−2Δη(P(η))Δη(P(η))T].

From Equation (A5), we know that $H_{\eta^{*}}\left(G\left(\eta \right)\right)⪯\Gamma \left(\eta^{*}\right)H_{\eta^{*}}\left(P\left(\eta \right)\right)$, and based on the fact that ${\left[\Delta_{\eta }\left(R_{\eta }\right)\right]}_{\eta =\eta^{*}}=0$, we can get:

(A8) $$\Delta_{\eta }\left(G\left(\eta \right)\right)\;=\;G\left(\eta \right)\;\left(\frac{U\;-\;M}{1\;-\;P(\eta )}\;-\;\frac{M\;-\;1}{P(\eta )}\right)\;\Delta_{\eta }\left(P\left(\eta \right)\right).$$

Substitute Equations (A5) and (A8) in Equation (A7), the Hessian matrix is simplified as:

(A9) $${\left[H_{\eta }\left(R_{\eta }\right)\right]}_{\eta =\eta^{*}}\;⪯\;\frac{-G(\eta^{*})}{P(\eta )(1-P(\eta ))}\Delta_{\eta^{*}}\left(P\left(\eta \right)\right)\Delta_{\eta^{*}}{\left(P\left(\eta \right)\right)}^{T}.$$

Since $\Delta_{\eta^{*}}\left(P\left(\eta \right)\right)\Delta_{\eta^{*}}{\left(P\left(\eta \right)\right)}^{T}$ is semidefinite positive, ${\left[H_{\eta }\left(R_{\eta }\right)\right]}_{\eta =\eta^{*}}⪯0$. Therefore, follow the fact that ${\left[\Delta_{\eta }\left(R_{\eta }\right)\right]}_{\eta =\eta^{*}}=0$ and ${\left[H_{\eta }\left(R_{\eta }\right)\right]}_{\eta =\eta^{*}}⪯0$, $\eta^{*}$ is locally optimal for the original optimization problem (Equation (22)). From Equation (A5), we know that $H_{\eta^{*}}\left(G\left(\eta \right)\right)⪯\Gamma \left(\eta^{*}\right)H_{\eta^{*}}\left(P\left(\eta \right)\right)$, and based on the fact that ${\left[\Delta_{\eta }\left(R_{\eta }\right)\right]}_{\eta =\eta^{*}}=0$, we can get:

(A10) $$\Delta_{\eta }\left(G\left(\eta \right)\right)\;=\;G\left(\eta \right)\;\left(\frac{U\;-\;M}{1\;-\;P(\eta )}\;-\;\frac{M\;-\;1}{P(\eta )}\right)\;\Delta_{\eta }\left(P\left(\eta \right)\right).$$

Substitute Equations (A5) and (A8) in Equation (A7), the Hessian matrix is simplified as:

(A11) $${\left[H_{\eta }\left(R_{\eta }\right)\right]}_{\eta =\eta^{*}}\;⪯\;\frac{-G(\eta^{*})}{P(\eta )(1-P(\eta ))}\Delta_{\eta^{*}}\left(P\left(\eta \right)\right)\Delta_{\eta^{*}}{\left(P\left(\eta \right)\right)}^{T}.$$

Since $\Delta_{\eta^{*}}\left(P\left(\eta \right)\right)\Delta_{\eta^{*}}{\left(P\left(\eta \right)\right)}^{T}$ is semidefinite positive, ${\left[H_{\eta }\left(R_{\eta }\right)\right]}_{\eta =\eta^{*}}⪯0$. Therefore, follow the fact that ${\left[\Delta_{\eta }\left(R_{\eta }\right)\right]}_{\eta =\eta^{*}}=0$ and ${\left[H_{\eta }\left(R_{\eta }\right)\right]}_{\eta =\eta^{*}}⪯0$, $\eta^{*}$ is locally optimal for the original optimization problem (Equation (22)). ☐
